# Advances in Deep-Learning-Based Sensing, Imaging, and Video Processing

**DOI:** 10.3390/s22166192

**Published:** 2022-08-18

**Authors:** Yun Zhang, Sam Kwong, Long Xu, Tiesong Zhao

**Affiliations:** 1 Shenzhen Institutes of Advanced Technology, Chinese Academy of Sciences, Shenzhen 518055, China; 2 Department of Computer Science, City University of Hong Kong, 83 Tatchee Ave., Kowloon, Hong Kong, China; 3 State Key Laboratory of Space Weather, National Space Science Center, Chinese Academy of Sciences, Beijing 100190, China; 4 College of Physics and Information Engineering, Fuzhou University, Fuzhou 350108, China

Deep learning techniques have shown their capabilities to discover knowledge from massive unstructured data, providing data-driven solutions for representation and decision making. They have demonstrated significant technical advancement potential for many research fields and applications, such as sensors and imaging, audio–visual signal processing, and pattern recognition. Today, with the rapid advancements of advanced deep learning models, such as conventional neural network (CNN), deep neural network (DNN), recurrent neural network (RNN), generative adversarial network (GAN), and transformer network, learning techniques, such as transfer learning, reinforcement learning, federal learning, multi-task learning, and meta-learning, and the increasing demands around effective visual signal processing, new opportunities are emerging in deep-learning-based sensing, imaging, and video processing. 

After a careful peer-review process, this editorial presents the manuscripts accepted for publication in the Special Issue “Advances in Deep-Learning-Based Sensing, Imaging, and Video Processing” of *Sensors*, which includes fourteen articles. These articles are original research papers describing current challenges, innovative methodologies, technical solutions, and real-world applications related to advances in deep-learning-based sensing, imaging, and video processing. They can generally be divided into two categories.

The first category is the deep-learning-based image and video processing by exploiting low-level visual features, including five articles [1,2,3,4,5]. Inspired by biological structure of avian retinas, Zhao et al. [1] developed a chromatic LED array with a geometric arrangement of multi-hyper uniformity to suppress frequency aliasing and color misregistration. The proposed concept provides insights for designing and manufacturing future bionic imaging sensors. To enhance image quality of imaging systems, Wang et al. [2] developed a novel color-dense illumination adjustment network (CIANet) for removing haze and smoke from fire scenario images. Schiopu et al. [3] explored a novel filtering method based on deep attention networks for the quality enhancement of light field (LF) images captured by plenoptic cameras and compressed by the high efficiency video coding (HEVC) standard. Tian et al. [4] proposed a dynamic neighborhood network (DNet) to dynamically select the neighborhood for local region feature learning in point clouds which improved the performances of point cloud classification and segmentation tasks. To access visual quality of videos, Lin et al. [5] proposed a no-reference objective video quality metric called saliency-aware artifact measurement (SAAM), which consists of an attentive CNN-LSTM network for video saliency detection, Densenet for distortion type classification, and support vector regression for quality prediction. These works reveal that deep learning models can exploit low-level visual features and promote imaging, image/video enhancement, segmentation, and quality assessment.

The second category relates to deep-learning-based visual object detection and analysis by exploiting higher-level visual and cognitive features. It contains nine articles [6,7,8,9,10,11,12,13,14]. Li et al. [6] developed a wheat ear recognition method based on RetinaNet and transfer learning by detecting the number of wheat ears as an essential indicator. This method can be used for automatic wheat ear recognition and yield estimation. To detect surface defects with variable scales, Xu et al. [7] proposed a multi-scale feature learning network (MSF-Net) based on a dual module feature (DMF) extractor, which classified the surface defects with multifarious sizes. In addition, Yu et al. [8] developed a deep-learning-based automatic pipe damage detection system for pipe maintenance. This detection system was composed of a laser-scanned pipe’s ultrasonic wave propagation imaging (UWPI) and CNN-based object detection algorithms. To inspect condition of hull surfaces by using underwater images acquired from a remotely controlled underwater vehicle (ROUV), Kim et al. [9] proposed a binary classification method by resembling multiple CNN classifiers which were transfer-learned from larger natural image datasets. Kim et al. [10] proposed a neg-region attention network (NRA-Net) to suppress negative areas and emphasize the texture information of objects in positive areas, which was then applied in an auto-encoder architecture based salient objects detection. He et al. [11] developed a small object detection algorithm named YOLO-MXANet for traffic scenes, which reduced the computational complexity of the object detection and meanwhile improved the detection accuracy. Alia et al. [12] proposed a hybrid deep learning and visualization framework of pushing behavior detection for pedestrian videos, which comprised a recurrent all-pairs field transforms (RAFT)-based motion extraction and an EfficientNet-B0-based pushing patches annotation. Deepfakes may cause information abuse by creating fake visual information. To verify video integrity, Lee et al. [13] presented a deep learning-based deepfake detection method by measuring changing rate of a number of visual features among adjacent frames. Then, a learned DNN was used to identify whether a video was manipulated. Xu et al. [14] proposed a timestamp-independent synchronization method for haptic–visual signals by exploiting a sequential cross-modality correlation between haptic and visual signals, where the deep learning network YOLO V3 was employed in visual object detection. In these works, deep learning technologies were applied to promote the performances of defect detection, object detection, anomaly detection, and recognition tasks in practical sensing, imaging, and video processing applications. 

We would like to thank all the authors and reviewers for their contributions to the Special Issue. We hope this Special Issue can provide some research insights, useful solutions, and exciting applications to scholars in academics and researchers in the industry interested in Deep-Learning-Based Sensing, Imaging, and Video Processing.

## Data Availability

Not applicable.
